# 512 Length of Stay Prediction Based on an Artificial Neural Network

**DOI:** 10.1093/jbcr/irae036.147

**Published:** 2024-04-17

**Authors:** Vishal Bandaru, Brandon Youssi, Ryan D D Morgan, Kevin M Nguyen, Xiyu Liu, Tristin Chaudhury, John A Griswold, Alan Pang

**Affiliations:** Texas Tech University Health Sciences Center, Austin, TX; Texas Tech University Health Sciences Center, Lubbock, TX; Texas Tech Health Science Center School of Medicine, Amarillo, TX; Texas Tech Health Sciences Center, Lubbock, TX; Texas Tech University Health Sciences Center, Austin, TX; Texas Tech University Health Sciences Center, Lubbock, TX; Texas Tech Health Science Center School of Medicine, Amarillo, TX; Texas Tech Health Sciences Center, Lubbock, TX; Texas Tech University Health Sciences Center, Austin, TX; Texas Tech University Health Sciences Center, Lubbock, TX; Texas Tech Health Science Center School of Medicine, Amarillo, TX; Texas Tech Health Sciences Center, Lubbock, TX; Texas Tech University Health Sciences Center, Austin, TX; Texas Tech University Health Sciences Center, Lubbock, TX; Texas Tech Health Science Center School of Medicine, Amarillo, TX; Texas Tech Health Sciences Center, Lubbock, TX; Texas Tech University Health Sciences Center, Austin, TX; Texas Tech University Health Sciences Center, Lubbock, TX; Texas Tech Health Science Center School of Medicine, Amarillo, TX; Texas Tech Health Sciences Center, Lubbock, TX; Texas Tech University Health Sciences Center, Austin, TX; Texas Tech University Health Sciences Center, Lubbock, TX; Texas Tech Health Science Center School of Medicine, Amarillo, TX; Texas Tech Health Sciences Center, Lubbock, TX; Texas Tech University Health Sciences Center, Austin, TX; Texas Tech University Health Sciences Center, Lubbock, TX; Texas Tech Health Science Center School of Medicine, Amarillo, TX; Texas Tech Health Sciences Center, Lubbock, TX; Texas Tech University Health Sciences Center, Austin, TX; Texas Tech University Health Sciences Center, Lubbock, TX; Texas Tech Health Science Center School of Medicine, Amarillo, TX; Texas Tech Health Sciences Center, Lubbock, TX

## Abstract

**Introduction:**

Hospital length of stay (LOS) is difficult to predict in patients with burn injuries due to the complexity of the injuries and the wide variability in patient outcomes. Predicting hospital LOS in patients with burn injuries can have numerous benefits for patients, physicians, and insurance companies. Artificial neural networks may offer insight into more accurate LOS predictions.

**Methods:**

We obtained electronic health records (EHR) for burn patients from July 01, 2011 - July 01, 2021. An artificial neural network was used to model the relationship between the input features and LOS. The input features were preprocessed by normalizing each feature to the range [0, 1] and transformed using log normal. The preprocessed data was split into training (70%), validation (15%), and testing (15%) sets. The neural network consisted of one hidden layer with 13 neurons and a linear output layer.

**Results:**

The neural network achieved an R-squared (R2) value of 0.72 on the training data, indicating that 72% of the variance in the data (n=1347). The validation and testing R2 values were 0.66 and 0.68, respectively. The overall R2 value, computed by combining the predictions on the training, validation, and testing data, was 0.70. The best performance, validation performance, and testing performance achieved by the neural network during the training process were 0.28, 0.27, and 0.30, respectively. These values indicate the average squared difference between the predicted output and the true output for each set, with lower values indicating better performance.

**Conclusions:**

The results suggest that the neural network can model the relationship between the input features and LOS with reasonable accuracy. The R2 values indicate that the model explains a substantial amount of the variability in the data. Further testing and validation may be needed to assess the robustness and reliability of the model.

**Applicability of Research to Practice:**

Patient outcomes are largely variable and determining LOS to a closer degree helps planning more robust treatment plans. Additionally, these models have the potential to serve as a more accurate gauge of whether they are following closely to their expected treatment timeline. As survival rates continue to improve, the improvement focus has and will continue to shift towards patient outcomes.

